# Attentive Prototype Learning with Wearable Sensor Mutual Information for Fall Risk Stratification of Parkinson’s Patients

**DOI:** 10.3390/bioengineering13060621

**Published:** 2026-05-26

**Authors:** Meng Zhang, Xuliang Ren, Jing Xu, Zhifen Guo, Qiumin Qu, Dongzhen Chen, Hongmei Cao

**Affiliations:** 1 Department of Neurology, The First Affiliated Hospital of Xi’an Jiaotong University, Xi’an 710061, China; zmeng8815@xjtu.edu.cn (M.Z.); 13324596249@163.com (Z.G.); quqiumin@xjtufh.edu.cn (Q.Q.); 2 School of Materials Science & Engineering, Key Laboratory of Functional Textile Material and Product of the Ministry of Education, Xi’an Polytechnic University, Xi’an 710048, China; 241321037@stu.xpu.edu.cn (X.R.); 241321036@stu.xpu.edu.cn (J.X.)

**Keywords:** fall risk quantification, Parkinson’s patients, attentive prototype learning, wearable sensors, biomechanical information

## Abstract

Parkinson’s disease (PD), with its rising global prevalence, poses severe risks from falls and motor impairments. Current fall risk assessments rely heavily on subjective clinical evaluations, underscoring the need for quantitative methods. In this exploratory study, wearable inertial and photoelectric sensors attached to the limbs and trunk were used to objectively collect biomechanical movement data during standardized MDS-UPDRS motor assessments. Leveraging the clinically validated correlation between Hoehn-Yahr (H-Y) staging and fall risk, we propose a data-driven framework to quantify risk. Mutual information (MI) analysis links biomechanical features to H-Y stages, generating a weighted Fall FRS (FRS). Machine learning validation was further performed to preliminarily evaluate the discriminative capability of the proposed FRS in stratifying patients by risk severity. Based on a cohort of 92 PD patients, experimental results on the independent test set showed that incorporation of the FRS improved classification accuracy from 50.00% to 82.14%, while the macro-average AUC increased from 0.698 to 0.907. These findings suggest that wearable sensor–based biomechanical assessment may provide useful quantitative information for exploratory fall-risk stratification in PD patients.

## 1. Introduction

At present, the incidence of Parkinson’s disease (PD) is gradually increasing. This will seriously affect the quality of life of patients. Falls, as a common problem for PD patients [1], have consequences including injuries, hospitalization, decreased mobility, and reduced quality of life [2]. Existing studies have shown that approximately 60% of PD patients have experienced at least one fall [3]. Compared with those who do not fall frequently, people with PD are more afraid of falling, have balance disorders, and experience motor fluctuations [4]. Current research indicates that gait and postural variability measurements are key factors in assessing the risk of falls [5]. Slower gait, increased walking variability [6], and postural instability are associated with an increased likelihood of falling [7,8]. Currently, the assessment of a patient’s fall risk usually relies on the subjective judgment of clinicians [9]. It is hoped that there will be a more objective, data-driven method to quantify the risk of falls and obtain the Fall FRS (FRS). This score functionally reflects a patient’s potential propensity for falls. By quantifying this FRS, it provides objective evidence for identifying patients with poor balance, thereby supporting the development of more targeted prevention and care strategies in clinical practice.

Wearable sensors are an increasingly popular method for identifying movement problems in PD patients [10]. Analyzing gait and posture data from wearable sensors can distinguish PD patients at different stages of the disease and identify movement symptoms such as tremors and gait freezing [11]. Existing studies have shown that patients with Parkinson’s disease exhibiting postural instability and gait disorders demonstrate significantly poorer balance capabilities [12]. Therefore, wearable sensors can be employed to analyze gait characteristics, thereby enabling quantitative estimation of balance abilities [13]. Existing studies have shown that the data obtained through continuous digital measurement, such as walking variability and the increased risk of falls, have a strong correlation [14]. This highlights the importance of continuous digital measurement. Although wearable-sensor-based assessment has shown considerable promise in Parkinson’s disease, several important limitations remain [15]. First, many existing studies rely on long-term continuous monitoring or free-living gait acquisition, which may limit clinical practicality and increase deployment complexity. Whether short-duration, standardized clinical motor assessments can provide sufficient information for quantitative fall-risk estimation remains insufficiently explored. Second, most previous studies mainly focused on binary fall classification or symptom identification, while relatively few studies attempted to construct an interpretable continuous fall-risk representation associated with disease progression. Third, many artificial intelligence-based approaches emphasize classification performance but provide limited interpretability regarding the contribution of individual biomechanical features to fall risk estimation. Therefore, developing an objective, interpretable, and clinically feasible framework for quantitative fall-risk assessment using short-term wearable sensor data remains an important unmet need. However, it remains unknown whether shorter, standardized, and clinically based gait and balance data can be used to quantitatively estimate the risk of falls in patients.

This study aims to quantitatively assess the risk of falls in patients with PD based on the data collected from wearable sensors during clinical short-term tests. Existing medical evidence has confirmed that the Hoehn-Yahr (H-Y) staging, as a classic quantitative indicator for the progression of PD, is significantly correlated with an increase in the risk of falls in patients. This provides an important clinical anchor for quantifying the risk of falls. To construct an interpretable quantitative representation of fall risk, it is essential to evaluate the relative contribution of different biomechanical features to disease progression and fall-related motor impairment. Compared with conventional linear correlation methods, mutual information can effectively capture both linear and nonlinear dependencies between variables without requiring predefined distribution assumptions [16,17]. This characteristic is particularly suitable for Parkinson’s disease-related biomechanical signals, in which gait abnormalities and balance impairments may exhibit complex nonlinear relationships. Furthermore, mutual information provides an interpretable quantitative measure of feature relevance, enabling weighted integration of multiple kinematic features into a unified fall-risk representation. Based on the calculated mutual information values, an individual FRS was subsequently constructed through feature-weighted fusion [18]. To systematically verify the rationality and discriminative value of this FRS, we constructed downstream machine learning models for validation analysis [19,20], ultimately achieving the verification of the effectiveness of the FRS.

## 2. Materials and Methods

### 2.1. Testing Task

**Figure 1:**
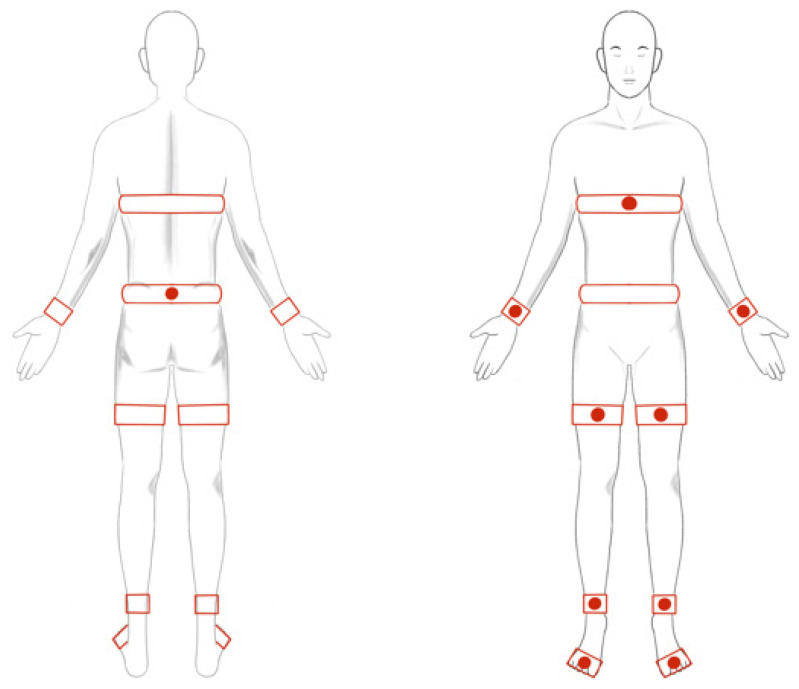
Placement configuration of the wearable inertial measurement unit (IMU) sensors used for gait and motion assessment in patients with Parkinson’s disease. A total of 10 IMU sensors were attached bilaterally to the wrists, thighs, shanks, and feet, with additional sensors positioned on the sternum and lumbar region. The sensor system was used to collect kinematic data during the Timed Up and Go (TUG) test. The red box represents the placement location of the wearable sensors on the human body.

To ensure the validity of data collection, all participants completed the tests under the condition of regular medication (i.e., during the medication period). Although this operation may underestimate the actual risk of falls in PD patients, it has the advantage of standardizing the drug exposure conditions, thereby preserving the relative differences in fall risk among patients at different disease stages. The effectiveness of quantifying fall risk through risk scoring is not affected. A wearable motion and gait quantification assessment system, MATRIX (GYENNO SCIENCE, Shenzhen, China), which is commercially available, was utilized in this study [21]. It is approved by the National Medical Products Administration (NMPA), U.S. Food and Drug Administration (FDA), and Conformitè Europëenne Medical (CEMedical). All participants were equipped with 10 IMU sensors, with a sampling rate of 100 Hz. Each IMU provided inertial sensing results via a (1) tri-axial accelerometer (range = ±16 g, sensitivity = 16,384 LSB/g) and a (2) tri-axial gyroscope (range = ±2000 dps, sensitivity = 131 LSB/dps). Two hand sensors were bilaterally placed on the dorsal side of the wrist. The chest sensor was placed on the sternum of the chest, and the waist sensor was attached to the fifth lumbar vertebra. Two thigh sensors were bilaterally placed 7 cm above the knee, while two shank sensors were bilaterally placed 7 cm below the knee. Two-foot sensors were bilaterally placed at the instep (dorsal side of the metatarsus) of each foot. All sensors were tightened to designated locations by straps (Figure 1). The TUG test was performed. During the TUG test, participants were instructed to stand up from a chair, walk in a straight line for 5 m at a comfortable speed, turn 180° at the end marker, walk back to the starting point, turn 180° in front of the chair, and sit down again. The total walking distance during the TUG assessment was 10 m.

### 2.2. Quantification of Fall Risk Based on Mutual Information Method

We calculate the FRS as a derived feature of the sample. To prevent the leakage of test set information, we divide it into a separate test set (30%) and a training set (70%). All feature engineering operations are strictly based on the parameters of the training set. We use Min-Max normalization to scale the features of the training set to the interval [0, 1], and the test set uses the standardizer fitted with the training set for transformation. The mutual information method is used to obtain the weights of different features in calculating the FRS (w) through the training set, and the weights obtained from the training set are used in calculating the FRS (w) of a single sample in the test set. The mutual information method is based on the principles of information theory and can objectively and unbiasedly quantify the association strength between features and the H-Y staging. Its calculation only relies on the data distribution itself and does not require preset assumptions such as linear or normal distribution, which can naturally capture the common nonlinear associations in clinical data and ensure the data-driven rationality of the weights. To enhance the stability of weight estimation under small-sample conditions, the training data undergoes multiple sampling via a Bootstrap resampling strategy during training. Feature mutual information values are computed after each sampling iteration. The results from multiple samples are ultimately averaged and normalized, ensuring the sum of all feature weights equals 1. This approach preserves the relative importance of features while guaranteeing scale consistency during multi-feature aggregation, providing a data-driven basis for stable calculation of FRSs. Previous studies have demonstrated a significant correlation between H-Y staging and patient balance ability [1,4,12]. Therefore, using H-Y staging as an intermediate variable and quantifying the association strength between features and H-Y staging via mutual information provides a reasonable approach to indirectly characterize FRS.

For both the training and test sets, step-length variability and stride variability were first extracted to construct the baseline gait-instability component. These two variables were selected because previous studies have demonstrated their close association with postural instability and fall tendency in Parkinson’s disease. The baseline gait-instability component was calculated by averaging the normalized values of step-length variability and stride variability for each sample. Specifically, both features were first normalized using the minimum and maximum values estimated from the training set, and the baseline component was then obtained as the arithmetic mean of the two normalized gait-variability measures. This baseline term was designed to provide a coarse representation of overall gait instability prior to the incorporation of feature-weighted deviation information in the final FRS formulation. The baseline component was derived exclusively from the training data and used as an initial representation of overall gait instability, rather than as a direct manual encoding of H-Y stages. After baseline construction, the remaining gait features were retained for subsequent risk-score calculation. To ensure consistency between training and testing procedures, all features were normalized using parameters estimated from the training set only. Based on the normalized training data, the global mean value of each feature was calculated as a reference distribution mean. For each sample, the deviation between its feature value and the corresponding training-set global mean was then computed to quantify the degree of gait deviation in each feature dimension. During testing, the normalization parameters and reference feature means obtained from the training set were directly reused without recalculation. This design prevents information leakage from the test set and improves the generalizability of the proposed framework. The resulting feature-deviation term reflects how individual gait characteristics deviate from the overall training distribution and provides a stable quantitative reference for subsequent fall-risk estimation.

To characterize the direction of influence of feature changes on disease severity, this study introduces a feature direction coefficient $d_{i}$ for each feature. Calculate the Pearson correlation coefficient $r_{i}$ between features and disease staging labels in the training set, and determine the direction based on its sign. When $r_{i}<0$, set $d_{i}=-1$; when $r_{i}>0$, set $d_{i}=1$. This ensures that feature contributions are consistent with the direction of disease progression. This strategy ensures that feature contributions align with the direction of disease progression during score accumulation.

Based on this, the feature weights $w_{i}$ and feature direction coefficients $d_{i}$ obtained through the mutual information method are weighted and summed to construct the FRS for each individual sample. Specifically, the baseline score $y_{j}$ is first assigned based on the sample’s H-Y stage, reflecting the fundamental level of overall motor function across different disease stages. Subsequently, calculate the difference $x_{ij}-u_{i}$ between each feature of the sample and the global mean $u_{i}$ of the training set. Combine the feature weights $w_{i}$ and feature directions $d_{i}$ obtained through mutual information calculations to perform a weighted summation of the deviations for each feature. Ultimately, by accumulating the contribution of all feature deviations based on the baseline score, the ${FRS}_{j}$ for an individual sample is obtained, enabling a comprehensive quantification of the patient’s risk. This scoring framework integrates weighted biomechanical feature deviations to provide a quantitative characterization of balance impairment and fall-risk severity in patients with Parkinson’s disease. Here, the feature weight reflects the relative importance of different gait features across disease stages, while the feature direction coefficient describes the positive or negative impact of feature changes on disease severity. This approach ensures the final score objectively reflects variations in gait stability. The quantitative formula for FRS in a single sample is:

$${FRS}_{j}=y_{j}+\sum_{i=1}^{n}w_{i}d_{i}(x_{ij}-u_{i})$$

Here, ${FRS}_{j}$ denotes the FRS for the jth sample; $y_{j}$ represents the baseline gait-severity component of the jth sample; n is the number of features involved in the score calculation; $w_{i}$ is the normalized weight of the ith feature calculated via the mutual information method; $d_{i}$ is the direction coefficient of the ith feature. $x_{ij}$ denotes the normalized value of the jth sample on the ith feature; $u_{i}$ represents the global mean of the ith feature within the training set. Through this approach, the baseline component provides a coarse representation of overall gait impairment severity, while the feature deviation term reflects the degree of variation in individual gait features relative to the overall reference pattern, thereby achieving a comprehensive quantification of the patient’s balance capability status.

### 2.3. Model Construction and Rationality Verification of FRS

**Figure 2:**
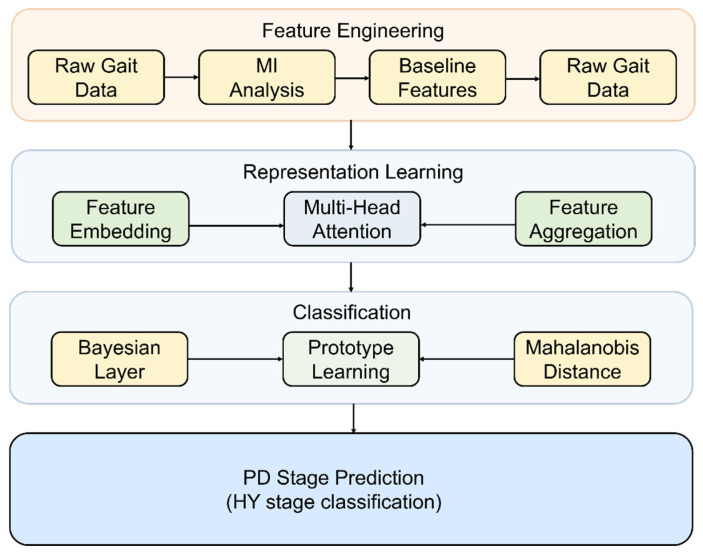
Overview of the proposed framework for Parkinson’s disease (PD) Hoehn–Yahr (H-Y) stage prediction using wearable sensor–derived gait features. First, gait biomechanical features are processed through feature engineering and Fall FRS (FRS) construction, where baseline gait-severity components, mutual information–based feature weights, and feature-deviation terms are integrated to generate a quantitative fall-risk representation. Subsequently, lightweight feature representation learning is performed through embedding and multi-head self-attention–based feature interaction modeling. The learned representations are then fed into a Bayesian and prototype-based classification module incorporating uncertainty-aware regularization and Mahalanobis distance-based prototype similarity. Finally, the integrated framework outputs H-Y stage predictions for quantitative assessment of PD motor severity and fall-related gait impairment.

This study constructs a classification model to evaluate the validity of the proposed FRS, with its framework illustrated in Figure 2. The proposed framework was intentionally designed as a lightweight feature-learning architecture suitable for small-sample biomechanical data analysis. The model integrates a shallow embedding module, a lightweight multi-head self-attention mechanism [22], Bayesian regularization, and prototype-based classification to perform lightweight feature interaction modeling while reducing overfitting risk. The model takes extracted gait features and the constructed FRS as inputs to evaluate whether the FRS contains information reflecting Parkinson’s disease severity and fall risk, through lightweight feature interaction modeling and prototype-similarity-based classification. During the feature representation phase, the input gait features are first fed into a shallow embedding module, which maps the original features into a compact representation space. This process improves feature expressiveness while maintaining low model complexity.

Subsequently, during the feature relationship modeling stage, a multi-head self-attention mechanism is introduced to capture latent interactions among different gait features. A lightweight multi-head self-attention mechanism was introduced to capture inter-feature relationships among gait variables. Unlike large-scale sequence modeling architectures, the attention module in this study employed only a limited embedding dimension and attention-head configuration to avoid excessive model capacity under small-sample conditions. The output from the attention module undergoes further fusion with the feature aggregation module via residual connections, ultimately yielding high-level feature representations for each sample.

During the classification stage, the model employs a Bayesian linear classifier to perform probabilistic modeling on the learned feature representations. Unlike traditional deterministic classifiers, Bayesian linear layer was primarily introduced as a regularization-oriented component to improve uncertainty modeling and reduce overfitting risk in small-sample training scenarios, thereby better capturing uncertainty in the prediction process. Additionally, Prototype learning was incorporated to encourage intra-class feature compactness by constraining latent representations toward category-specific feature centers, thereby improving representation stability. Specifically, a feature prototype vector is learned for each category. The similarity between a sample and different categories is measured by calculating the Mahalanobis distance between the sample representation and each category’s prototype, thereby enabling the final classification decision.

During model training, a stratified sampling strategy was employed to partition the dataset into training and testing sets at a 7:3 ratio. To mitigate class imbalance, the Synthetic Minority Oversampling Technique (SMOTE) oversampling was applied exclusively to the training set after data partitioning and was never used on the independent test set, thereby preventing information leakage during model evaluation. Model optimization employs the Adam optimizer for training. To improve reproducibility and reduce model instability, fixed random seed initialization, feature standardization, dropout regularization, residual normalization, and early stopping strategies were employed during training. Early stopping was triggered when the training loss failed to improve within a predefined patience window. The loss function combines cross-entropy loss with a KL divergence regularization term, where the KL divergence constrains the posterior distribution of Bayesian parameters to enhance the model’s generalization capability.

Two control models were designed for the study. One model included the FRS as an input feature, while the other model excluded this score. By comparing the differences in key indicators (accuracy rate, macro-average AUC, and class precision rate) between the two models, the enhancing effect of the score on the model performance was quantified. If the performance of the model including the score was significantly better than that of the model excluding the score, it would confirm its clinical rationality and discriminative value. Considering the relatively limited sample size and class imbalance across H-Y stages, the present framework should be interpreted as a preliminary exploratory investigation rather than a clinically validated deployment model. Further validation using larger multi-center cohorts will be necessary to confirm the robustness and generalizability of the proposed framework.

The detailed implementation settings of the proposed model are summarized in Table 1, ensuring full reproducibility of the experimental setup.

## 3. Results

Based on the clinical short-term test data collected by wearable sensors, our aim was to quantify the risk of patient falls [23]. The sample distribution was 10 patients in stage 1, 13 patients in stage 1.5, 25 patients in stage 2, 34 patients in stage 3, and 10 patients in stage 4. Since the participants included patients with high H-Y stages, to ensure the safety of the patients and the reliability of the data, the data collected restricted that the medication status of all patients must be on (i.e., in the best state of pH-Ysical condition) [24]. In the end, 92 participants met the inclusion criteria for the analysis. The patients included in this study ranged in age from 48 to 73 years.

### 3.1. The Walking Variability Performance Serves as the Benchmark for Quantifying the Risk of Falling

First, a total of 25 gait-related features were extracted from wearable sensor data (Table 2). Among these, stride length variability and stride length variation exhibited a monotonically increasing trend across different H-Y phases, and were thus employed to construct the baseline score. The remaining features were assigned normalized mutual information weights based on their association with H-Y classification, and their respective feature directions were determined according to their correlation signs.

**Figure 3:**
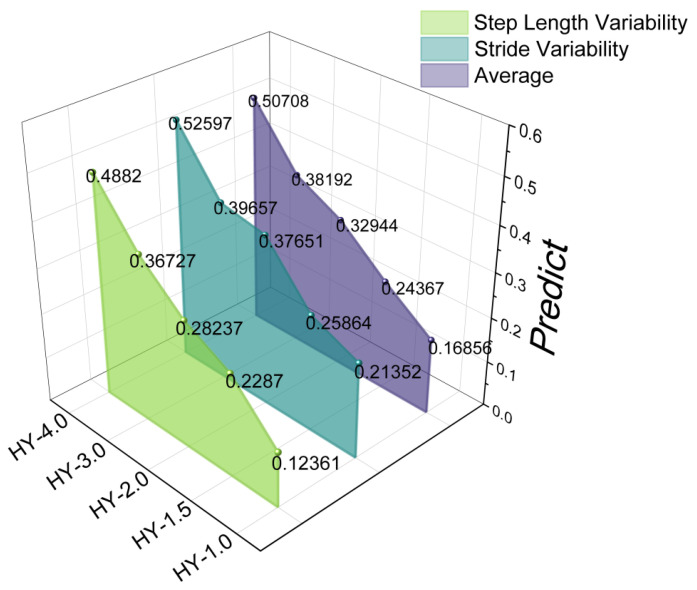
Group-wise distributions of step length variability and stride length variability across H-Y stages. The figure presents the within-stage mean values of both gait features after outlier removal and normalization to reduce scale differences. In addition, a fused mean trend, calculated as the average of the two gait features within each H-Y stage, is included to summarize the overall pattern of gait variability.

After outlier removal, the group-wise mean values of step length variability and stride length variability were computed for each H-Y stage. To reduce scale differences between features, the group means were normalized using their global extrema. In addition to the stage-wise feature means, the overall mean trends of both features across H-Y stages were also calculated to provide a reference-level comparison of progression patterns. The results show that both gait features, as well as their corresponding mean trends, exhibit a consistent increasing pattern with disease progression, as illustrated in Figure 3.

**Figure 4:**
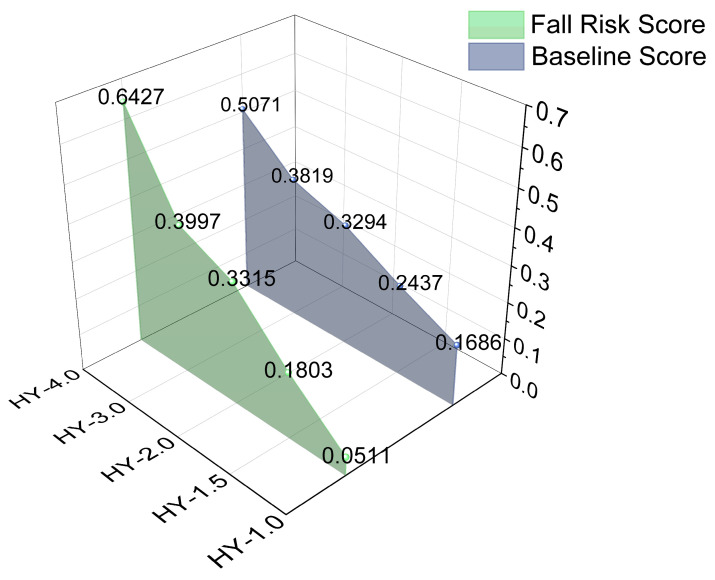
Trends of baseline score and fall FRS across Hoehn–Yahr (H-Y) stages. The baseline score is derived from gait variability features representing overall motor impairment, while the fall FRS is constructed using a mutual information–based feature weighting strategy. Both scores are normalized for comparison across stages. Although both indicators show a consistent increasing trend with disease progression, the fall FRS is lower than the baseline score in early H-Y stages (1–1.5) and becomes higher in later stages (≥2), while both exhibit similar overall progression patterns across stages.

Both step-length variability and stride variability exhibited progressively increasing trends across H-Y stages, indicating worsening gait instability with disease progression. A marked increase was observed from stage 3 to stage 4, suggesting a transition toward more pronounced motor impairment. The baseline scores and fall FRSs across different H-Y stages are presented in Figure 4.

### 3.2. Verify the Effectiveness of the FRS Through Machine Learning Models

To investigate whether the constructed FRS encodes information associated with fall risk, the input data were divided into two experimental settings: (1) A training set and a test set without the derived FRS; (2) A training set and a test set including the derived FRS.

**Figure 5:**
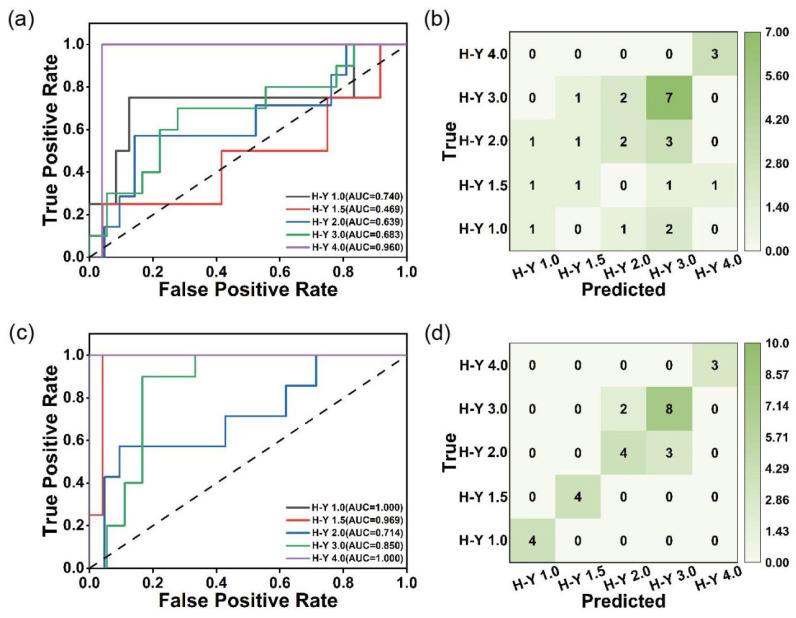
(**a**,**b**) present the five-class ROC curves and confusion matrix of the model trained without the FRS on the independent test set, respectively. (**c**,**d**) show the corresponding ROC curves and confusion matrix of the model trained with the FRS. The results demonstrate the performance difference between the two experimental settings in classifying the five H-Y stages.

The evaluation results of the models trained on the two sets of data on the test set are shown in Figure 5. Without the inclusion of the FRS feature, the ROC curve results are presented in Figure 5a. The AUC values for H-Y 1.0, H-Y 1.5, H-Y 2.0, H-Y 3.0, and H-Y 4.0 were 0.740, 0.469, 0.639, 0.683, and 0.960, respectively. These results indicate that the model exhibited strong discriminative ability only for the H-Y 4.0 stage, whereas its performance in distinguishing the early-to-middle stages, particularly H-Y 1.5 and H-Y 2.0, remained limited, suggesting insufficient sensitivity to early disease progression in the absence of FRS information. The corresponding confusion matrix is shown in Figure 5b, where all three samples in the H-Y 4.0 stage were correctly classified. However, only two samples in the H-Y 2.0 stage were correctly identified, while the remaining samples were mainly misclassified as H-Y 3.0. Similarly, only one sample in the H-Y 1.5 stage was correctly predicted, with misclassifications distributed across H-Y 1.0, H-Y 3.0, and H-Y 4.0. In addition, although seven samples in the H-Y 3.0 stage were correctly classified, several samples were still misclassified as H-Y 1.5 and H-Y 2.0, indicating considerable overlap among adjacent disease stages. After incorporating the FRS feature, the model performance improved markedly. As shown in Figure 5c, the AUC values increased to 1.000 for H-Y 1.0, 0.969 for H-Y 1.5, 0.714 for H-Y 2.0, 0.850 for H-Y 3.0, and 1.000 for H-Y 4.0. Compared with the model without FRS, the AUC value for the H-Y 1.5 stage increased substantially from 0.469 to 0.969, while that for H-Y 2.0 increased from 0.639 to 0.714, indicating that the FRS feature effectively enhanced the model’s sensitivity to differences among the early-to-middle stages of disease progression. The corresponding confusion matrix is presented in Figure 5d, where both the H-Y 1.0, H-Y 1.5 and H-Y 4.0 stages achieved 100% classification accuracy. All eight samples in the H-Y 3.0 stage were correctly predicted, with only a few H-Y 2.0 samples being misclassified into this stage. Meanwhile, the number of correctly classified H-Y 2.0 samples increased from two to four, demonstrating improved classification stability. These findings demonstrate that the incorporation of the FRS feature significantly improves the model’s ability to distinguish different PD stages, particularly for patients in the early and intermediate transitional stages.

Detailed per-class classification metrics are summarized in Table 3. In addition, Table 3 presents a comparison of per-class performance metrics (precision, recall, specificity, and F1-score) across H-Y stages with and without the proposed FRS. The results indicate an overall improvement in classification performance after incorporating the FRS, with more pronounced gains observed in intermediate stages (H-Y 2.0–3.0), where class overlap is more significant.

**Figure 6:**
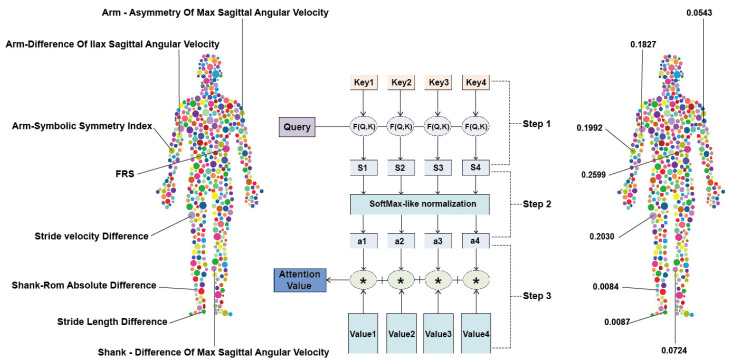
Visualization of the top eight gait features ranked by attention-based interaction strength. The multi-head self-attention module captures pairwise dependencies among gait features, and the displayed weights reflect the relative strength of feature interactions within the learned representation space.

All evaluation metrics in Table 3 were computed from the confusion matrices illustrated in Figure 5b,d. Overall, incorporating the proposed FRS improved the classification performance of the model, enhanced feature discriminability, and improved classification balance while reducing false prediction errors. Although most evaluation metrics exhibited an overall improvement trend after introducing the FRS, several localized fluctuations were still observed across different H-Y stages. In particular, the F1-score for H-Y stage 2.0 decreased from 0.857 (without the FRS) to 0.615 (with the FRS). This phenomenon may be related to the relatively overlapping gait characteristics and limited sample size within intermediate disease stages, where small variations in prediction distribution can substantially influence per-class metrics. These findings suggest that the proposed FRS does not uniformly optimize every individual metric for all stage-specific subsets, but instead primarily improves the global discriminative structure of the learned feature representation space. Despite these localized fluctuations, the proposed FRS still provides meaningful discriminative information and enhances model robustness under small-sample conditions. The confusion matrix results further demonstrate that incorporating the FRS improves classification consistency across different disease stages, particularly for intermediate H-Y stages with relatively overlapping gait patterns. To further examine the role of the proposed FRS, the attention-based feature interaction weights were visualized in Figure 6. Among the top eight features ranked by interaction strength, the FRS shows relatively higher values compared with other gait variables, indicating stronger interactions within the learned feature representation space. It is important to clarify that the FRS is not intended as a direct predictor of model performance. In this study, attention weights are used to reflect feature interaction strength rather than feature importance, indicating how strongly the FRS interacts with other gait variables in the learned representation space.

## 4. Discussion

Fall risk in patients with Parkinson’s disease (PD) was assessed using data collected from wearable sensors. This study is based on data-driven approaches to objectively quantify the risk of falls, without involving subjective human will. The aim is to investigate the ability of kinematic data to quantify the risk of falls. When obtaining the kinematic data, the medication status of all patients was in the open period. During the process of evaluating the effectiveness of the FRS, we divided the data into two groups: one containing the FRS and the other not containing it, for ablation experiments. We used the Bayesian prototype network as the verification model and combined the multi-head attention mechanism to learn the feature interaction relationships [25,26]. By comparing the ability of the models trained with the two sets of data to identify the H-Y stages of different PD patients, we demonstrated the effectiveness of the FRS.

When evaluating the classification performance of the model, the attention mechanism scores were extracted from the model trained using the data set containing the FRS [27]. This observation indicates that the FRS contributes meaningful discriminative information within the learned feature space. The FRS has been calculated and its validity has been verified. These findings may provide preliminary insights for future clinical decision support research. Several treatment plans and strategies for preventing falls have been proposed [28,29], which focus on exercise [30,31], fall prevention education and complex nursing interventions [32,33]. The key protection targets should be patients whose FRS is significantly higher than the average level of their corresponding H-Y stage.

The main limitation of this study lies in the limited sample size. The quantification of FRS relies on statistical methods, and the stability and accuracy of such methods are inherently constrained by the sample size. The bias in the distribution of features in a small sample may lead to estimation errors in the weights of the scores, thereby affecting the reliability of risk quantification. Although the independent test set is the core basis for verifying the generalizability of the model, after the data set was divided in this study, the sample size of some categories in the test set was only 3. Such a small subsample size is difficult to support robust statistical model evaluation, which may lead to incorrect judgments on the generalization performance of the model. We derived new features named FRS for each sample based on the kinematic data of the samples. The FRS is essentially used to measure the deterioration of multiple balance abilities of patients, and its value is calculated through a weighted summation method. The limitation of the data volume may lead to over-assigning or neglecting the weights of some features, but with the optimization of feature derivation techniques, such weight deviations can be gradually alleviated. The design concept of this study also provides an expandable methodological reference and research direction for related fields.

Beyond data scale, the model architecture itself may also influence results. This study employs a classification framework integrating attention mechanisms, Bayesian learning, and prototype learning. The attention mechanism captures latent interactions among different gait features, while prototype learning enhances class discriminative power by learning class centers, thereby contributing to model stability under small-sample conditions. However, deep models may still face overfitting risks with limited samples, and parameter uncertainty could affect final classification outcomes. Therefore, future research should validate these models on larger datasets to assess their generalization capabilities and stability across varying data distributions.

Despite the promising results, the proposed framework may still be subject to overfitting due to the limited sample size and the use of relatively expressive model components, including attention-based feature modeling and Bayesian parameterization. Although regularization strategies such as early stopping, normalization, and constrained architecture design were employed, the risk of model instability under small-sample conditions cannot be fully eliminated.

One limitation of this study is that the dataset does not include detailed clinical information on Parkinson’s disease subtypes (e.g., idiopathic, age-related onset, or familial PD), which prevented subtype-specific analyses. However, the proposed method focuses on biomechanical gait characteristics derived from wearable sensor data rather than etiological differences, and therefore this limitation is unlikely to substantially affect the overall conclusions. Future studies should incorporate datasets with more comprehensive clinical information to further investigate potential differences in gait patterns and fall-risk characteristics among PD subtypes.

Therefore, future research should focus on significantly increasing the sample size, especially by supplementing the samples of low-frequency categories. This will enhance the statistical power of the data set. This not only reduces the bias in the estimation of feature distribution but also provides a more solid empirical basis for the cross-population generalization of FRS and the reliability of model evaluation, thereby enhancing the reference value of research conclusions in clinical practice. The current research focuses on quantifying the fall risk of PD patients, which is helpful for formulating personalized care and medical measures for different PD patients. Studies have shown that wearable sensors and machine learning can be used to identify potential PD patients with high fall risk at each H-Y stage. Short-term gait and posture data can be used to develop long-term care strategies for PD patients. The design of the model is based on two main goals: Firstly, through ablation experiments to compare the performance of models trained with different data to verify the effectiveness of the FRS. Secondly, by making the data processing methods transparent to clinical experts, we ensure the reliability of the model. Identifying potential high fall-risk patients may pave the way for more targeted and effective PD management strategies, ultimately helping to prevent life-threatening falls.

## 5. Conclusions

This study presents a wearable sensor–based framework for fall risk assessment in Parkinson’s disease by constructing a weighted FRS from gait features. The results indicate that the proposed FRS improves the discriminative performance of downstream machine learning models, suggesting that gait-derived composite indicators may contain informative representations related to disease progression.

While the findings support the feasibility of the proposed approach, the study remains exploratory due to the limited sample size, and further validation using larger external cohorts is necessary to confirm its robustness and clinical applicability.

## Figures and Tables

**Table 1:** Model implementation details including data preprocessing, feature-based architecture configuration, H-Yperparameters, training strategy, and reproducibility settings for the proposed Parkinson’s disease fall risk classification framework.

Category	Parameter	Value
Data preprocessing	Feature scaling	StandardScaler (fitted on training set only)
	Train-test split	Predefined fixed split
	Data leakage control	SMOTE applied only on training set
Input features	Input dimension	Determined by selected gait feature set
Architecture	Model structure	Embedding → Multi-head Attention → Bayesian Linear → Prototype Layer → Classifier
	Number of modules (layers)	Four feature learning modules with a dual-head prediction structure
Embedding layer	Output dimension	64
Attention module	Type	Multi-head self-attention
	Number of heads	4
	Dropout	0.1
	Residual connection	Yes
Bayesian layer	Type	Bayesian Linear layer
	KL divergence weight	1 × 10^−5^
Prototype learning	Distance metric	Mahalanobis distance
	Class prototypes	Learnable per class
Classifier	Type	Linear layer
Training setup	Optimizer	Adam
	Learning rate	1 × 10^−3^
	Batch size	8
	Maximum epochs	200
Early stopping	Metric monitored	Training loss
	Patience	15 epochs
	Delta threshold	1 × 10^−4^
Loss function	Objective	Cross-entropy + KL divergence regularization
Reproducibility	Random seed	42
	Deterministic mode	Enabled (torch.backends.cudnn.det erministic=True)
	Benchmark mode	Disabled
Hardware environment	Device	GPU (CUDA-enabled if available)
	Framework	PyTorch

**Table 2:** The extracted 25 gait features along with their corresponding mutual information values, normalized weights, feature directions, and functional roles in FRS construction. Stride variability and gait cycle M variability represent different gait variability measures and are treated as independent variables in this study.

Feature	Weight (The Normalized Mutual-Information Weights)	Direction	Role
SteptimeVariability	0.0519	Positive	Feature
Phase Coordination Index	0.0517	Positive	Feature
SteptimeAsym	0.0517	Positive	Feature
GaitCycleMVariability	0.0505	Positive	Feature
Mean Phase Difference	0.0504	Positive	Feature
Stride Length Asymmetry	0.0488	Positive	Feature
Shank-RoM Absolute Difference	0.0477	Negative	Feature
Shank-RoM Asymmetry	0.0476	Positive	Feature
Stride Length Difference	0.0458	Negative	Feature
Arm-Difference Of Max Sagittal Angular Velocity	0.0457	Negative	Feature
Stride Velocity Asymmetry	0.0456	Positive	Feature
Shank-Symbolic Symmetry Index	0.0430	Positive	Feature
Arm-Symbolic Symmetry Index	0.0417	Positive	Feature
Arm-Asymmetry Of Max Sagittal Angular Velocity	0.0407	Negative	Feature
Shank-Asymmetry Of Max Sagittal Angular Velocity	0.0406	Positive	Feature
Swing Asymmetry	0.0394	Positive	Feature
Step Asymmetry	0.0392	Positive	Feature
Stride Velocity Difference	0.0382	Negative	Feature
StanceAsymm	0.0366	Positive	Feature
Shank-Difference Of Max Sagittal Angular Velocity	0.0364	Negative	Feature
Stance Asymmetry	0.0363	Positive	Feature
Stance Absolute Difference	0.0353	Positive	Feature
Swing Absolute Difference	0.0353	Positive	Feature
Stride Variability	0	Positive	Baseline (Y_1_)
StepLenVariability	0	Positive	Baseline (Y_2_)

**Table 3:** Summarizes the quantitative results of models equipped with or without FRS under diverse H-Y stages. It can be found that embedding FRS achieves obvious optimization on mainstream evaluation indicators, yet several metrics exhibit different changing tendencies without universal enhancement.

H-Y Stage	Precision	Recall (Sensitivity)	Specificity	F1-Score	Include the FRS
H-Y 1.0	1.000	1.000	1.000	1.000	Yes
H-Y 1.5	1.000	1.000	1.000	1.000	
H-Y 2.0	0.667	0.571	0.905	0.615	
H-Y 3.0	0.727	0.800	0.833	0.762	
H-Y 4.0	1.000	1.000	1.000	1.000	
H-Y 1.0	0.333	0.250	0.286	0.917	No
H-Y 1.5	0.333	0.250	0.286	0.917	
H-Y 2.0	0.400	0.286	0.333	0.857	
H-Y 3.0	0.539	0.700	0.609	0.667	
H-Y 4.0	0.750	1.000	0.857	0.960	

## Data Availability

Due to privacy concerns, the data generated and analyzed in this study will not be made public. However, upon reasonable requests from the corresponding authors, such data can be provided to them.

## References

[1] Fasano A., Canning C.G., Hausdorff J.M., Lord S., Rochester L. (2017). Falls in Parkinson’s disease: A complex and evolving picture. Mov. Disord..

[2] Kurth T., Brinks R. (2025). Projecting Parkinson’s disease burden. BMJ.

[3] Allen N.E., Schwarzel A.K., Canning C.G. (2013). Recurrent falls in Parkinson’s disease: A systematic review. Park. Dis..

[4] Wilczyński J., Ścipniak M., Ścipniak K., Margiel K., Wilczyński I., Zieliński R., Sobolewski P. (2021). Assessment of risk factors for falls among patients with Parkinson’s disease. BioMed Res. Int..

[5] Sotirakis C., Brzezicki M.A., Patel S., Conway N., FitzGerald J.J., Antoniades C.A. (2024). Predicting future fallers in Parkinson’s disease using kinematic data over a period of 5 years. npj Digit. Med..

[6] Lord S., Galna B., Yarnall A.J., Coleman S., Burn D., Rochester L. (2016). Predicting first fall in newly diagnosed Parkinson’s disease: Insights from a fall-naïve cohort. Mov. Disord..

[7] Mak M., Auyeung M. (2013). The mini-BESTest can predict parkinsonian recurrent fallers: A 6-month prospective study. J. Rehabil. Med..

[8] Lindholm B., Hagell P., Hansson O., Nilsson M.H. (2015). Prediction of falls and/or near falls in people with mild Parkinson’s Disease. PLoS ONE.

[9] Pickering R.M., Grimbergen Y.A., Rigney U., Ashburn A., Mazibrada G., Wood B., Gray P., Kerr G., Bloem B.R. (2007). A meta-analysis of six prospective studies of falling in Parkinson’s disease. Mov. Disord..

[10] FitzGerald J.J., Lu Z., Jareonsettasin P., Antoniades C.A. (2018). Quantifying motor impairment in movement disorders. Front. Neurosci..

[11] Reichmann H., Klingelhoefer L., Bendig J. (2023). The use of wearables for the diagnosis and treatment of Parkinson’s disease. J. Neural Transm..

[12] Pelicioni P.H.S., Menant J.C., Latt M.D., Lord S.R. (2019). Falls inParkinson’s disease subtypes: Risk factors, locations and circumstances. Int. J. Environ. Res. Public Health.

[13] Hasegawa N., Shah V.V., Carlson-Kuhta P., Nutt J.G., Horak F.B., Mancini M. (2019). How to select balance measures sensitive to Parkinson’s disease from body-worn inertial sensors—Separating the trees from the forest. Sensors.

[14] Weiss A., Herman T., Giladi N., Hausdorff J.M. (2014). Objective assessment of fall risk in Parkinson’s disease using a body-fixed sensor worn for 3 days. PLoS ONE.

[15] Fu X., Ni S., Al-qaness M.A. (2026). Parkinson’s disease detection based on artificial intelligence: Methodologies, datasets, clinical applications, challenges and future directions. Eng. Appl. Artif. Intell..

[16] Golub P., Antalik A., Beran P., Brabec J. (2023). Mutual information prediction for strongly correlated systems. Chem. Phys. Lett..

[17] Huang L., Zhou X., Shi L., Gong L. (2024). Time series feature selection method based on mutual information. Appl. Sci..

[18] Zhang P., Liu G., Song J. (2023). MFSJMI: Multi-label feature selection considering join mutual information and interaction weight. Pattern Recognit..

[19] Sigcha L., Borzì L., Olmo G. (2024). Deep learning algorithms for detecting freezing of gait in Parkinson’s disease: A cross-dataset study. Expert Syst. Appl..

[20] Yin W., Zhu W., Gao H., Niu X., Shen C., Fan X., Wang C. (2024). Gait analysis in the early stage of Parkinson’s disease with a machine learning approach. Front. Neurol..

[21] GYENNO Technologies CO. Ltd (2022). GYENNO MATRIX-Wearable Motion and Gait Quantitative Evaluation System. https://www.gyenno.com/matrix-en.

[22] Li C., Gong P., Li S., Tian C., Yu Y., Wang R., Zhang D., Zhu Q. (2026). Spatio-Temporal Hypergraph Attention Networks for Brain Disease Analysis. IEEE Trans. Image Process..

[23] Ullrich M., Roth N., Kuderle A., Richer R., Gladow T., Gasner H., Marxreiter F., Klucken J., Eskofier B.M., Kluge F. (2022). Fall risk prediction in Parkinson’s disease using realworld inertial sensor gait data. IEEE J. Biomed. Health Inform..

[24] Conklin S.J., Cavalcanti H.M., Almeida L.R.S., Mishra V., Oliveira-Filho J., Mari Z., Landers M.R., Longhurst J.K. (2025). Identifying gait characteristics associated with freezing of gait in Parkinson’s disease: An analysis of on and off medication states. Gait Posture.

[25] Li C., Liu M., Yan X., Teng G. (2022). Research on CNN-BiLSTM fall detection algorithm based on improved attention mechanism. Appl. Sci..

[26] Sang H.F., Chen Z.Z., He D.K. (2020). Human motion prediction based on attention mechanism. Multimed. Tools Appl..

[27] Sun Y., Pang S., Qiu Z., Zhang Y. (2025). Efficient lithology classification from small-sample well logging data processed by wavelet thresholding algorithm: Integrating meta-learning with self-attention mechanism model. Geoenergy Sci. Eng..

[28] Canning C.G., E Allen N., Bloem B.R., Keus S.H., Munneke M., Nieuwboer A., Sherrington C., Verheyden G.S. (2022). Interventions for preventing falls in Parkinson’s disease. Cochrane Database Syst. Rev..

[29] Canning C.G., Paul S.S., Nieuwboer A. (2014). Prevention of falls in Parkinson’s disease: A review of fall risk factors and the role of pH-Ysical interventions. Neurodegener. Dis. Manag..

[30] Sherrington C., Michaleff Z.A., Fairhall N., Paul S.S., Tiedemann A., Whitney J., Cumming R.G., Herbert R.D., Close J.C., Lord S.R. (2017). Exercise to prevent falls in older adults: An updated systematic review and meta-analysis. Br. J. Sports Med..

[31] E Morris M., Taylor N.F., Watts J.J., Evans A., Horne M., Kempster P., Danoudis M., McGinley J., Martin C., Menz H.B. (2017). A home program of strength training, movement strategy training and education did not prevent falls in people with Parkinson’s disease: A randomised trial. J. Physiother..

[32] Dent E., Martin F.C., Bergman H., Woo J., Romero-Ortuno R., Walston J.D. (2019). Management of frailty: Opportunities, challenges, and future directions. Lancet.

[33] A Logan P., Horne J.C., Gladman J.R.F., Gordon A.L., Sach T., Clark A., Robinson K., Armstrong S., Stirling S., Leighton P. (2021). Multifactorial falls prevention programme compared with usual care in UK care homes for older people: Multicentre cluster randomised controlled trial with economic evaluation. BMJ.

